# Feedback Between Behavioral Adaptations and Disease Dynamics

**DOI:** 10.1038/s41598-018-30471-0

**Published:** 2018-08-20

**Authors:** Jiangzhuo Chen, Achla Marathe, Madhav Marathe

**Affiliations:** 1 Network Dynamics and Simulation Science Laboratory, Virginia Tech, Blacksburg, VA 24061 USA; 2 Department of Agricultural and Applied Economics, Virginia Tech, Blacksburg, VA 24061 USA; 3 Department of Computer Science, Virginia Tech, Blacksburg, VA 24061 USA

## Abstract

We study the feedback processes between individual behavior, disease prevalence, interventions and social networks during an influenza pandemic when a limited stockpile of antivirals is shared between the private and the public sectors. An economic model that uses prevalence-elastic demand for interventions is combined with a detailed social network and a disease propagation model to understand the feedback mechanism between epidemic dynamics, market behavior, individual perceptions, and the social network. An urban and a rural region are simulated to assess the robustness of results. Results show that an optimal split between the private and public sectors can be reached to contain the disease but the accessibility of antivirals from the private sector is skewed towards the richest income quartile. Also, larger allocations to the private sector result in wastage where individuals who do not need it are able to purchase it but who need it cannot afford it. Disease prevalence increases with household size and total contact time but not by degree in the social network, whereas wastage of antivirals decreases with degree and contact time. The best utilization of drugs is achieved when individuals with high contact time use them, who tend to be the school-aged children of large families.

## Introduction

Successful pandemic preparedness for influenza demands full participation from the private and public sectors. A coordinated effort by business, industry and public sector is essential to minimize the economic and health impacts of a pandemic. Private sector is key to limiting the impact on the economy since it controls the supply chain of critical goods, logistical operations and personnel. The public sector ensures that all critical infrastructures and resources stay operational and accessible.

Antivirals are likely to play an important role in containing a severe influenza pandemic such as the one in 1918^1–4^. Although well matched vaccines are very useful, they are unlikely to be available during the early phase of a pandemic when a novel influenza strain emerges. Antivirals combined with social distancing-based interventions have been proposed as a part of federal and global strategies to contain the pandemic and decrease mortality until a vaccine becomes available. Public health agencies have studied the problem of vaccine manufacturing and distribution since the early 1990s but distribution of antivirals has received lesser attention. Potential benefits of prophylactic antiviral drugs have created a renewed interest in assessing the antiviral drug use strategies and potential stockpiling targets by the private and public sectors during a pandemic^5–8^.

This research considers the distribution of a limited antiviral stockpile between the markets (private sector) and the hospitals (public sector) with the aim of meeting the following goals: keep the disease spread under control by treating the infected at the hospitals for free; sell the market stockpile at a nominal cost so the worried-well at home can buy the antivirals for prevention; and help recover the cost of the stockpile. The market stockpile can also be used for treatment but since antivirals are available free of cost from the hospitals, they only purchase it for prevention until the hospital stockpile runs out, at which point the market stockpile is used for treatment as well. The demand for antivirals in the market is driven by the price, individuals’ budget, and the level of disease prevalence. As the disease dynamics change over time, the preventive demand for antivirals creates interesting market dynamics as well as disease dynamics. A higher level of disease prevalence increases the preventive demand for antivirals in the market and vice versa. Individuals can also decide to “stay home” if the disease prevalence is high.

The emergent macro behavior from this setting explains some well known phenomenon in the economics of epidemiology^9,10^, e.g. the oscillatory behavior of disease prevalence. When the prevalence is high, the demand for prevention is high which together with a smaller pool of susceptibles, makes the disease self limiting. However, when the prevalence is low, the demand for prevention is low, which makes it progressively harder to eradicate the disease.

This research explains how changes in individual behavior affect and are affected by the spread of infectious diseases. A multi-layer, multi-theory modeling environment studies the feedback processes between disease dynamics, preventive behavior, and social distancing and its effect on the social contact network, when a limited supply of antivirals is available. We identify optimum strategies that divide the antiviral stockpile between the market and the hospitals so that the epidemic stays under control and the entire cost of antivirals is recovered through the market. This is combined with a social distancing strategy that modifies the social network and changes opportunities for transmission, eventually affecting the evolution of the disease^11^.

*Novelty:* This paper makes a number of novel contributions: (*i*) It uses a micro analysis framework to study macro phenomenon. Explains the cause and effect and feedback between interventions, market constraints, disease prevalence, and contact networks; (*ii*) Studies an urban (Washington DC) and a rural region (New River Valley of Southwest Virginia); (*iii*) Uses network properties and demographics to find who carries the highest burden of disease and who can afford antivirals from the market; (*iv*) Uses a network measure to define and assess wastage of market stockpile; (*v*) Determines if there is an interaction between wastage of resources, demographics, and network properties; and (*vi*) Studies the sensitivity of results to a variety of parameters.

## Related Work

The US Homeland Security Council on National Strategy for Pandemic Influenza^4^ states that “Private stockpiles, in coordination with public health stockpiles, would extend protection more broadly than could be achieved through the public sector alone and improve the ability to achieve the national pandemic response goals of mitigating disease, suffering, and death, and minimizing impacts on the economy and functioning of society.” As the Centers for Disease Control and Prevention (CDC) considers alternative distribution methods for antivirals through private systems during a pandemic, an Association of State and Territorial Health Officials (ASTHO) report^3^ highlights the need to answer the following question: how should CDC decide to breakdown the stockpile among private and public distributors? This study takes a step in answering this question.

Previous researchers^12–17^ have considered many related aspects of this problem. For example, Althouse *et al*.^15^ consider market-based distribution of antivirals during an influenza pandemic but only for treatment purposes. With their parameter settings, a market-based distribution results in over or under-use of antivirals relative to the efficient level. Too few people buy it if required to purchase in advance of the pandemic and too many people buy it if allowed to purchase at the time of infection. Goldstein *et al*.^8^ examine the benefits of pre-dispensing antivirals under a variety of scenarios including the case when demand exceeds supply.

Our research focuses on building a detailed individual-based causal model and a micro economic framework to study emergent macro behaviors and disease dynamics. It considers both market and public sector based distribution of antivirals, and both treatment and preventive use of antivirals, along with the behavioral adaptations by individuals during the course of the pandemic.

A report by the US Department of Health and Human Services discusses conditions under which interested employers can stockpile antivirals^2^. The Institute of Medicine report recommends coordination and communication with the private sector on dispensing and distributing antivirals^1^. Wu *et al*.^18^ study the possible benefits of multidrug strategies over mono-therapy for reducing the impact of antiviral resistance. Acemoglu *et al*.^19^ provide a general discussion on the importance of using both markets and governments in resource allocation.

Other researchers have studied similar questions but in other contexts. For example, previous research^20–23^ studies the coevolution of friendship and smoking behavior under a variety of scenarios; Adams *et al*.^24^ show how edges made through sex-ties and drug-ties differentially contribute to observed network racial segregation; Mitleton-Kelly^25^ studies coevolution of intelligent social systems; Hammond *et al*.^26^ discuss feedback loops between agri-food, health, disease, and environmental systems; and Epstein *et al*.^27^ use an agent based model of interacting contagion processes, i.e. disease and fear, to study interaction between behavior and social networks in the event of epidemics.

However none of the studies consider detailed interactions between the market factors, preventive behavior, changes in the social contact network and epidemic outcomes, and their cause and effect on each other. This is the first study that explains the feedback loops between all these components.

## Methodology

We use a detailed individual-based, social contact network model to study the spread of a novel strain of influenza^28^. Two regions, the New River Valley (NRV) of Southwest Virginia and Washington DC (WDC) are considered. Their social networks are constructed using datasets explained in Table 1 (except the last two). The disease propagation follows SEIR (*Susceptible* → *Exposed* → *Infectious* → *Recovered*) model, as given in §3.2, and transmits infection as explained in the transmission model in §3.3. Interventions to mitigate the epidemic include antiviral administration that decreases the probability of an infectious individual transmitting the disease to other people and that of a susceptible individual getting infected by other people. The social distancing strategy removes edges from the social contact network and cuts off pathways to transmission. The interventions are explained in §3.4. We discuss the market demand of antivirals in §3.5 and a model for measuring the wastage of antivirals in §3.6. Finally §3.7 describes the multi-theory multi-layer representation of the modeling environment.

**Table 1:** List of Select Datasets.

Dataset	Description
US Census	**Source**: http://www.census.gov
	**Description**: US census data.
	**Usage**: Used in building synthetic populations with block group level statistics matched to US census.
National Household Travel Survey(NHTS)	**Source**: http://nhts.ornl.gov
	**Description**: Data on people’s activity and travel behavior.
	**Usage**: Used as an input to modeling activities of each person.
Dun & Bradstreet(D&B)	**Source**: http://www.dnb.com
	**Description**: Various locations and their capacities are described in this dataset.
	**Usage**: Used to model the locations at which activities take place.
American Time Use Survey(ATUS)	**Source**: http://www.bls.gov/tus
	**Description**: Survey data on people’s activities such as paid work, childcare, shopping, home etc.
	**Usage**: Used to model the activities of people.
NAVTEQ/HERE	**Source**: https://here.com/en/navteq
	**Description**: Road network map for the study regions.
	**Usage**: Used in calibrating activity time and locations for building the social network.
Disease Model	**Source** ^11, 34, 35^
	**Description**: Various parameters of the disease model.
	**Usage**: Used in building the disease model
Consumer Expenditure Survey	**Source**: http://www.bls.gov/cex/
	**Description**: Average health care budget for households.
	**Usage**: Used to model antiviral budget

### Models of Social Contact Networks

The experiments are conducted for two regions, WDC and NRV of Southwest Virginia. WDC is an urban region whereas NRV is mostly rural. We run computer simulations of disease propagation on the synthetic social contact networks of these regions. The nodes of the network represent people and edges represent interactions between people. The methodology used to generate the networks is described in^29–33^, and these networks have been used in a variety of public health policy studies e.g.^32,34–37^, and most recently in^38–41^.

**Figure 1:**
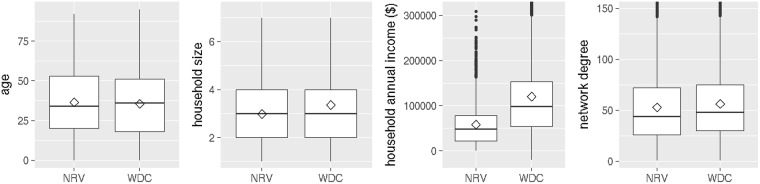
The box plots compare NRV and WDC population characteristics in terms of demographics and network properties. Distributions of age, household size, household income, and network degree are shown respectively. The diamond symbol in each box plot represents the mean of the variable.

WDC has 1.66 million households and its social network is comprised of 4.13 million nodes (people) and 233 million edges (contacts). The NRV social contact network is substantially smaller–it has 153 thousand nodes, 8.1 million edges and consists of 66 thousand households. Figure 1 compares the distributions of the demographic and network attributes between NRV and WDC.

### Disease Model

We use SEIR disease model to study the disease propagation during an influenza pandemic^34,42^. Each person in the model is in one of the following four health states at any given time: *susceptible*, *exposed*, *infectious*, and *recovered*.

- A person is in a susceptible health state until s/he becomes exposed.
- If person *v* becomes exposed, s/he remains exposed for Incub[v] days (called *incubation period*), during which s/he is not infectious.
- Then s/he becomes infectious and remains infectious for Infect[v] days (called *infectious period*), during which s/he may be *symptomatic* or *asymptomatic*. An asymptomatic person is less likely to transmit the disease to other people.
- Finally s/he becomes removed (or recovered) and remains so for the remaining time period.

When an individual becomes infectious, with probability 2/3 s/he becomes symptomatic and with probability 1/3 s/he remains asymptomatic. In the latter case s/he is 47% less likely to transmit the disease^43,44^; i.e., the probability of this individual transmitting the disease to any other individual (defined in the equation in §3.3) is reduced by 47%. The symptomatic individuals report to the hospital and are diagnosed with probability 0.60^11^. The diagnosed individuals are given one course of the antiviral until the hospital stockpile of antivirals runs out. The antivirals are given free of cost to the diagnosed individuals at the hospital. The model parameters and assumptions are similar to earlier influenza pandemic studies done by the NIH-MIDAS (Modeling of Infectious Disease Agent Study) group^11,34^.

### Transmission Model

An infectious node *u* transmits the disease to a susceptible node *v* with probability

$$p(w(u,v))=1-{(1-r)}^{w(u,v)}$$

where *r* is the probability of disease transmission for each unit of time of contact between *u* and *v*^39,45^. The unit of time is measured in minutes. In our networks, edge (*u*, *v*) with weight *w*(*u*, *v*) represents that node *u* has contact of duration *w*(*u*, *v*) with node *v*, during which the disease *may* transmit from an infectious node *u* to a susceptible node *v* with probability *p*(*w*(*u*, *v*)).

The spread of infection from node *u* to node *v* is assumed to be completely independent of the infection from node *u*′ to node *v*. Similarly, an infected node *u* spreads the infection to each neighbor *v*, independent of the other neighbors of *u*. This is a central assumption in almost all the epidemic models and the analytical results based on percolation^46^.

We assume a base attack rate (cumulative number of infections as a percentage of population) of 40% which is likely to occur during a pandemic when no intervention is applied^47^. The disease transmission rate *r* is calibrated to achieve this 40% attack rate, resulting in transmission rates of 5.5125e–05 and 6.375e–05 per minute of contact time for WDC and NRV regions respectively.

### Interventions

We use *antivirals* and *stay-home* as two interventions^5,34^ for controlling the pandemic. The market based demand for antivirals is a function of household specific characteristics such as income, budget, and family size. Demand is also a function of the market price of antivirals and the level of disease prevalence. The prevalence elastic demand for antivirals ties demand behavior to disease dynamics.

Market stockpile of antivirals can be used *both* prophylactically as well as for treatment but the hospital stockpile is available only for treatment. The symptomatic individuals first try and procure antivirals from the public stockpile–given that hospitals give it for free to those who are diagnosed. When the hospital stockpile runs out, everyone depends on the market solely.

The “stay-home” strategy is applied to the diagnosed individuals after overall level of disease prevalence reaches a threshold. This social distancing strategy changes the structure of the social contact network and hence opportunities for transmission. More details on the interventions are provided in §4.2.

### Market Demand Model

We explore distribution of antivirals through the market as was considered during H1N1 pandemic in countries like New Zealand and India^11^. We assume a fixed stockpile of antivirals or inelastic supply in the market but elastic demand for antivirals by households. The demand for antivirals by household *h* on day *t*, *D*_*t*,*h*_, is assumed to depend on the current level of disease prevalence *x*_*t*_, the market price *P*_*t*_, and the budget *B*_*t*,*h*_ i.e. *D*_*t*,*h*_ = *f*(*x*_*t*_, *P*_*t*_, *B*_*t*,*h*_). Usually $\frac{\partial f}{\partial {x}_{t}}\ge 0,\frac{\partial f}{\partial {P}_{t}}\le 0,\frac{\partial f}{\partial {B}_{t,h}}\ge 0$.

We consider two functional forms of the demand function, linear and exponential. In the linear case the demand is a linear function of disease prevalence *x*_*t*_ (fraction of people infectious on day *t*) i.e. ${D}_{t,h}=\frac{{B}_{t,h}}{{P}_{t}}(\alpha +\frac{{x}_{t}}{\beta })$, upper bounded by $\frac{{B}_{t,h}}{{P}_{t}}$. In the exponential case the demand is an exponential function of *x*_*t*_, ${D}_{t,h}=\frac{{B}_{t,h}}{{P}_{t}}\mathrm{(1}-{e}^{-\beta {x}_{t}})$. Each function represents a different risk preference towards the disease, as measured by the parameter *β*. Most of the analysis in this paper is focused on the exponential demand case.

### Model for Measuring Wastage of Antivirals

An antiviral is considered *wasted* if it does not help protect any susceptible person (reduce his/her vulnerability) or help prevent any infectious person from spreading (reduce his/her infectivity). An antiviral that is applied to person A through a hospital is never considered wasted since A gets it only if A is diagnosed; so it always helps prevent A from spreading.

An antiviral applied to a susceptible person A through the market is not considered wasted if either of the following is true during the *effective days* [*t*, *t* + *D*): (*i*) A becomes infectious during the effective days; or (*ii*) A has a contact who is in an infectious state during the effective days. Note that *t* is the day the antiviral is applied to A, and *D* is the length of the application. In our study we assume *D* = 10 days.

### Multi-theory Multi-layer Model

**Figure 2:**
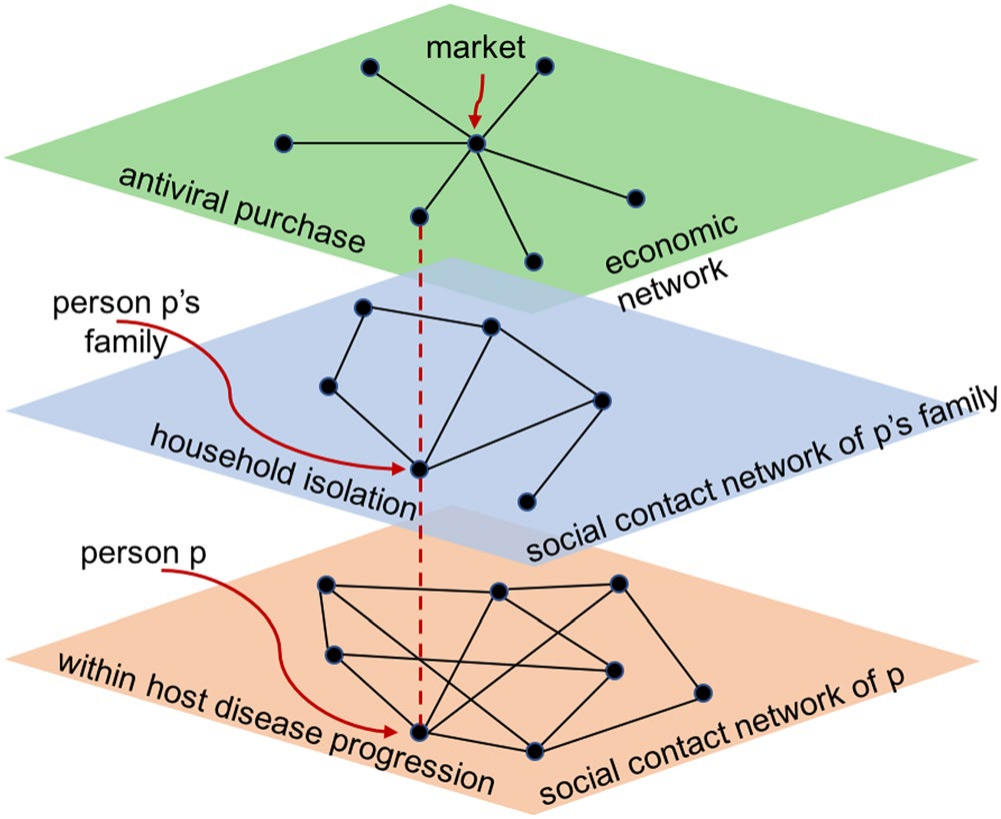
Multi-layer representation of multi-theory modeling environment. It shows how different network layers are influenced by behavioral adaptation. As disease propagation triggers household isolation and antiviral purchase, the price of the antiviral is impacted, the social contact network connectivity is affected, and consequently the opportunities for disease transmission are reduced.

Individual behavior, social network, and disease prevalence are highly interconnected and constantly feedback into each other. Without a detailed individual-based behavioral model, a social contact network, and a disease propagation model, interactions and feedbacks within and between components cannot be studied. This research builds a multi-layer representation of a multi-theory modeling environment, as shown in Fig. 2, to understand the continuously evolving dynamics among components^48–50^.

**Figure 3:**
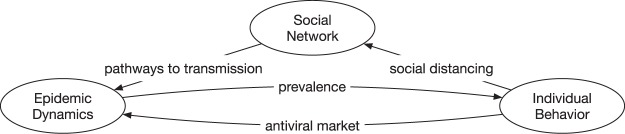
Feedback loop between epidemic dynamics, social network, and individual behavior. It shows how disease prevalence affects individual behavior, which in turn affects the social network, the market stockpile, and the market price, which in turn affect the disease prevalence.

The schematic in Fig. 3 shows the feedback loop between these components and the direction of influence.

- Epidemic dynamics ⇒ individual behavior. Disease prevalence makes individuals apply “stay-home” measure and/or buy antiviral drugs from the market.
- Individual behavior ⇒ social network. “Stay-home” intervention removes edges in the social contact network and hence reduces its connectivity.
- Social network ⇒ epidemic dynamics. Reduced connectivity of the social contact network reduces pathways to transmission which in turn affect the epidemic dynamics.
- Individual behavior ⇒ epidemic dynamics. Antivirals taken by individuals lower their vulnerability to the disease and their infectivity to other people, which in turn affect the disease prevalence, which in turn affect the demand for antivirals.

## Simulation Setup

This section describes our experimental and simulation setup. The simulation tool, data, and parameters are described below in detail.

### Simulation Tool

We use an individual based, discrete time simulator called **EpiFast**^51^ for simulating disease propagation on large social contact networks. It uses a parallel algorithm, which enables scaling on distributed memory systems. It is implemented in C++/MPI and is scalable to millions of agents. **EpiFast** belongs to a new emerging class of models called network-based epidemiological models that use a detailed representation of social contact networks; such a representation is crucial for studying questions related to the role of individual behavior and public policies. This highly disaggregated, agent based model can represent each interaction between individuals and hence study critical pathways of disease transmission. Disaggregate models require neither partitions of the population nor assumptions about large scale regularity of interactions. See^31,32,34,52,53^ for results and discussions on this topic.

### Experimental Setting

We assume that a limited supply of antivirals is available to cover about 10% of the population in each of the two regions^5^. This amounts to 15,000 units of antivirals in NRV and 400,000 in WDC. Each region’s stockpile is divided between the market and hospitals. The hospital stockpile is available for treating the diagnosed but the diagnosed have a compliance probability of 0.50, i.e. only half of the people will take the prescribed antivirals. The market stockpile is available for purchase to anyone who can afford it and can be used for either prevention or treatment.

Each household, observes daily the level of epidemic prevalence and the market price of antivirals, and determines its demand for the antivirals. If a household has just made a purchase and is still using the purchased antiviral, it has no demand. The antiviral dose lasts 10 days. The price of the antivirals depends on the remaining amount of supply: $${P}_{t}={P}_{{\rm{\max }}}-({P}_{{\rm{\max }}}-{P}_{{\rm{\min }}})\frac{{Q}_{t}}{{Q}_{0}}$$, where *P*_max_ and *P*_min_ are upper and lower bounds set by the government, and *Q*_*t*_ is the remaining antiviral supply in the market on day *t*. We set *P*_max_ = $100, *P*_min_ = $50, which includes the cost of producing and dispensing the antiviral^54^. The budget available for antiviral purchase is 1% of the household’s income. According to the consumer expenditure survey by the US Department of Labor, Bureau of Labor Statistics (http://www.bls.gov/cex/), the average health care budget is 6% of household income. We assume 15% of the health care budget is available for antiviral purchase during the pandemic. We use an exponential demand function and set *β* = 40.

The market purchase mechanism works as follows. If a household has a positive demand for an antiviral, it buys one course for each member, unless the market supply exhausts, or its budget is insufficient to buy one course per member. It may buy antivirals again after the previous course is over, until its budget for the antivirals is used up. Each course of antiviral is applied for 10 continuous days. When antiviral is applied to an infectious person, his/her probability of transmitting the disease is reduced by 80%^5^; when applied to a susceptible person, his/her probability of being infected is reduced by 87%^5,7,55^.

A social distancing measure, “stay-home”, is taken by individuals depending on their health states as well as the overall level of the disease prevalence. We assume the social distancing intervention does not trigger until the new infections on that day reach 0.05% of the population. After the threshold is met, when an individual is diagnosed as infectious, with compliance probability 0.50, he stays home until fully recovered.

The epidemic starts from 10 random seeds and is simulated for 300 days in each simulation run. For every experiment 30 replicates are created and the mean values are reported. Incubation and infectious period durations are chosen from distributions, with means equal to 1.9 and 4.1 days, respectively^34,35^. Different levels of split between the public (hospitals) and the private (markets) sectors are considered, starting from no antivirals to the markets, to all to markets.

To check the sensitivity of experimental results, we varied the settings of several parameters. The sensitivity analysis results are discussed in §6.

### Model validation and calibration

Extensive efforts have been made to validate the overall approach and specific components of the models. These include structural validity of models, matching the data produced to field data, and formal specifications of these models for software verification. For further information on validation of social contact network, we refer the reader to^29,31,32,34,38,39,56^. Models of within host disease progression and between host disease spread, parameters for the size of the antiviral stockpile, compliance to interventions, attack rate, price of antivirals etc. are taken from various sources in the literature as described in Table 2. Table 1 contains information on select data sources used to develop and calibrate our models.

**Table 2:** List of parameter values and their sources.

Parameter	Value	Source
Attack Rate or Cumulative Infection Rate	40–50%	^36, 47^
Transmission rate	calibrated to attack rate	not applicable
Proportion of symptomatic	0.67	^34, 44, 57^
Avg. Incubation period	1.9 days	^35^
Avg. Infectious period	4.1 days	^35^
Diagnosis probability	0.60	^34^
Compliance to Antiviral	0.50–0.75	^58^
Compliance to “Stay-home”	0.50–0.75	^59, 60^
Price of Antiviral (per course)	$50–100	^54^
Efficacy of Antiviral Preexposure	87%	^5, 55^
Efficacy of Antiviral Postexposure	80%	^55^
Antiviral Stockpile	10%	^5, 54^

## Results

A brief summary of results is given below.

1. We find that in both regions, it is beneficial to split the antiviral stockpile between the private and public sectors because it helps to control the spread of the disease and recover the cost of antivirals. However beyond a critical point, further allocation of the stockpile to market results in wastage of antivirals and a larger number of infections. In fact, the size of the epidemic increases with higher allocations to market. The prevalence-elastic demand of antivirals combined with the prevalence-based social distancing strategy of “stay-home” reduces and delays the peak of the epidemic and the overall size of the epidemic. Prevalence-based interventions also capture the oscillatory behavior of the epidemic.
2. Analysis of the burden of disease by demographics shows that the number of infections increase with family size and decrease with household income. In terms of network properties, infections increase with an individual’s total contact time but the relationship with degree is ambiguous. Higher degree does not always result in more occurrence of infection. However, wastage of market stockpile drops with network degree and total contact time.
3. We find that the best utilization of antivirals is when it is given to individuals who have the most contact time or the weighted degree. However in order to make this policy implementable, we searched for demographics that correlate with contact time. The findings show that school children, ages 5 to 18 years old, from large families have the highest contact time.

### Antiviral Allocation and Usage

We use different allocation fractions to split the antiviral stockpile between the market and the hospitals. We start with allocating all antivirals to the hospitals and none (0%) to the market, and then increase the market share in increments of 5% until the entire stockpile (100%) is allocated to the market.

**Figure 4:**
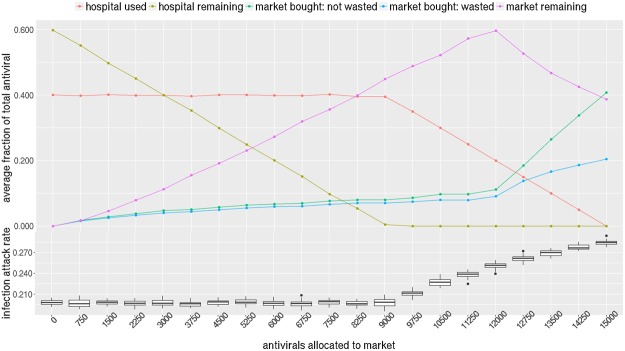
NRV has a stockpile of 15000 antivirals i.e 10% of the size of population. Horizontal axis shows the part of stockpile assigned to markets (remaining assigned to the hospitals). Vertical axis of the bottom plot shows the infection attack rate i.e. total number of infections as a fraction of the population; the top plot shows the average fraction of total antivirals used, wasted, and remaining in market/hospitals. After market allocation reaches 9000 or 60% of the stockpile, the infection attack rate shows a dramatic increase. This is explained by the fact that hospitals run out of their stockpile and increasing numbers of antivirals are either unused or wasted in the market.

Figure 4 shows how antiviral stockpiles are being used by the hospitals and market under different allocation fractions. The bottom part of Fig. 4 shows that epidemic size (or total number of infections) increases as higher amounts of antiviral are allocated to the market in NRV. The epidemic size increases rapidly when the market allocation is increased beyond 9000. Although it appears that the epidemic size is flat to the left of 9000, it is in fact gradually rising but the increase is statistically insignificant.

The optimal split between market and hospitals stands at 9000 (60%) and 6000 (40%) respectively. At this split level, the hospital stockpile is completely used up and there is nothing remaining from the hospital allocation. If less than 6000 is given to the hospitals, the hospitals are not left with enough to treat the diagnosed individuals (hospital remaining is 0) who then become dependent on the market. However everyone cannot afford to purchase from the market, which leaves infectious untreated and circulating. Note that the hospitals never use more than 6000 (40%) of the stockpile so allocating more to the hospital would not be helpful. Therefore it is better to give the remaining to the market to recover the cost of the antivirals.

The top part of Fig. 4 shows after 9000, as diagnosed individuals go untreated, the epidemic size starts to rise. As more and more of the stockpile is allocated to the market, the market stockpile either remains unused or wasted in the market. Wastage is measured as stated in §3.6. The “market remaining” increases because as the epidemic size increases, the demand for antivirals goes up since it is prevalence-elastic, forcing the price to increase faster, and making it unaffordable to households who want it. Note that the hospital stockpile is distributed for free to those who are diagnosed so there is no wastage of antivirals in the hospitals.

Similar pattern can be observed in WDC where a total stockpile of 400,000 (10% of population size) antivirals is available. The optimal split occurs at 300,000 to the market and 100,000 to the hospitals. One difference to note is that there is a lot more wastage in WDC. This is because household incomes are much higher in WDC (median $84,240) compared to NRV (median $40,000) (see Fig. 1), which means more households can afford to purchase at higher prices. This also results in a smaller “market remaining” portion.

**Figure 5:**
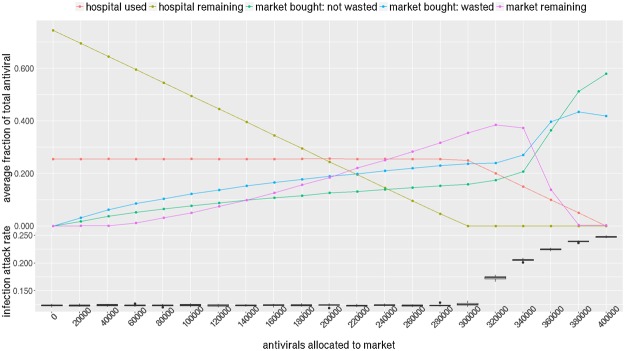
WDC has a stockpile of 400,000 antivirals i.e 10% of the size of population. Horizontal axis shows the part of stockpile assigned to markets (remaining assigned to the hospitals). Vertical axis of the bottom plot shows the infection attack rate i.e. total number of infections as a fraction of the population; the top plot shows the average fraction of total antivirals used, wasted, and remaining in market/hospitals. After market allocation reaches 300,000 or 75% of the stockpile, the infection attack rate shows a dramatic increase. This is explained by the fact that hospitals run out of their stockpile and increasing numbers of antivirals are either unused or wasted in the market.

Given the price range of *P*_max_ = $100, *P*_min_ = $50, an allocation of 9,000 to the market results in an average market price of $55.59 per course of antiviral and a total revenue of $0.126 million in NRV. In WDC, an allocation of 300,000 to the market results in an average market price of $62.70 per course and a total revenue of $9.923 million. Note that on average, only 25% of the market’s 9,000 allocation in NRV is actually purchased in the market, the rest remains unused; in WDC, this usage level is 53% as shown in Figs 4 and 5 respectively. This implies that as long as the cost of producing antiviral can be kept below $8.43 in NRV and below $24.81 in WDC, the revenue will be able to recover the total cost of the entire stockpile (market plus hospitals, used and remaining).

We check the sensitivity of the price cap by setting *P*_max_ to $75. The drop in the maximum price does not cause any significant change in the epidemic size, but increases the market usage marginally and drops the average market price in both NRV and WDC.

Note that this methodology can be applied to other regions with other experimental or real-world settings. Quantitative values such as average price, total revenue, and break-even cost may vary when alternative populations and parameters are considered. However the qualitative results and trends are expected to remain similar. Novelty of this work lies in the fact that it integrates a detailed social network model and a disease propagation model, with an economic model of interventions and behaviors to study disease dynamics. This level of detailed modeling has never been done before.

### Feedback Between Disease Dynamics, Behaviors and Social Networks

**Figure 6:**
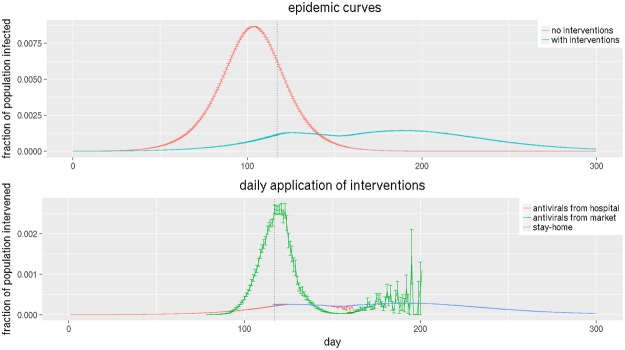
The bottom part shows the fraction of population intervened in WDC. “Antivirals from hospital” represents antivirals taken from the hospital; “antivirals from market” represents antivirals purchased from the market; and “stay-home” refers to individuals staying home after they are diagnosed to be infected. The top plot shows the epidemic curves or the fraction of population that gets infected by day. The red curve shows infections for the base case when no interventions are applied, and the turquoise curve shows infections when interventions as shown in the bottom part are applied. The curves show mean values of 30 replicates and the bars show one standard error above and below the mean values.

Next we explain how the disease, individual behavior, and social network evolve through a continuous feedback loop. Figure 6 explains how interventions are triggered by the spread of the disease and the dynamics of the disease is influenced by the interventions. There are two types of interventions considered here; antivirals and social distancing. Antivirals affect the susceptibility and infectivity of nodes whereas social distancing blocks pathways to transmission by removing edges in the social network.

The bottom part of the plot shows the fraction of population to whom interventions are applied and the top plot shows the fraction of population that gets infected with and without interventions. The perforated vertical line shows the time at which “stay-home” intervention is triggered which is set at 0.05% prevalence level. In other words, when disease prevalence in the region reaches 0.05%, those who are diagnosed as infected choose, with a compliance probability of 0.50, to stay home until they recover.

The epidemic curve with interventions shows oscillatory behavior and captures how disease prevalence moves in sync with the interventions. In this bimodal curve, the first peak occurs just after the “stay-home” intervention starts; until then, only antivirals were being used. The hospital stockpile starts to be utilized early on but the market stockpile is used only after prevalence rises enough to create demand as modeled by the demand function. However the efficiency of the market stockpile in controlling the epidemic is low since a lot of it is either wasted or remains unused.

“Stay-home” intervention starts to show its impact on the infections; the epidemic turns around but soon after the hospital stockpile runs out around day 170, the infections start to climb back up again, reaching another peak at around day 200. The exact days when this phenomenon is observed are sensitive to the selection of the simulation parameters but the trends remain similar. In the current setting, note that between day 150 and 200, the demand for market stockpile is very low. This is because the prevalence is high so the demand is high, but at the same time the market inventory is running low which raises the price, making it affordable to only a few. At about day 200, the market stockpile runs out and the only intervention in effect is “stay-home”. The epidemic curve moves perfectly in sync with the “stay-home” curve from this point onwards. The curves show mean values of 30 replicates and the bars show one standard error above and below the mean values. Please see the sensitivity section §6 for details on parameter sensitivity.

### Burden of Disease by Subpopulation

**Figure 7:**
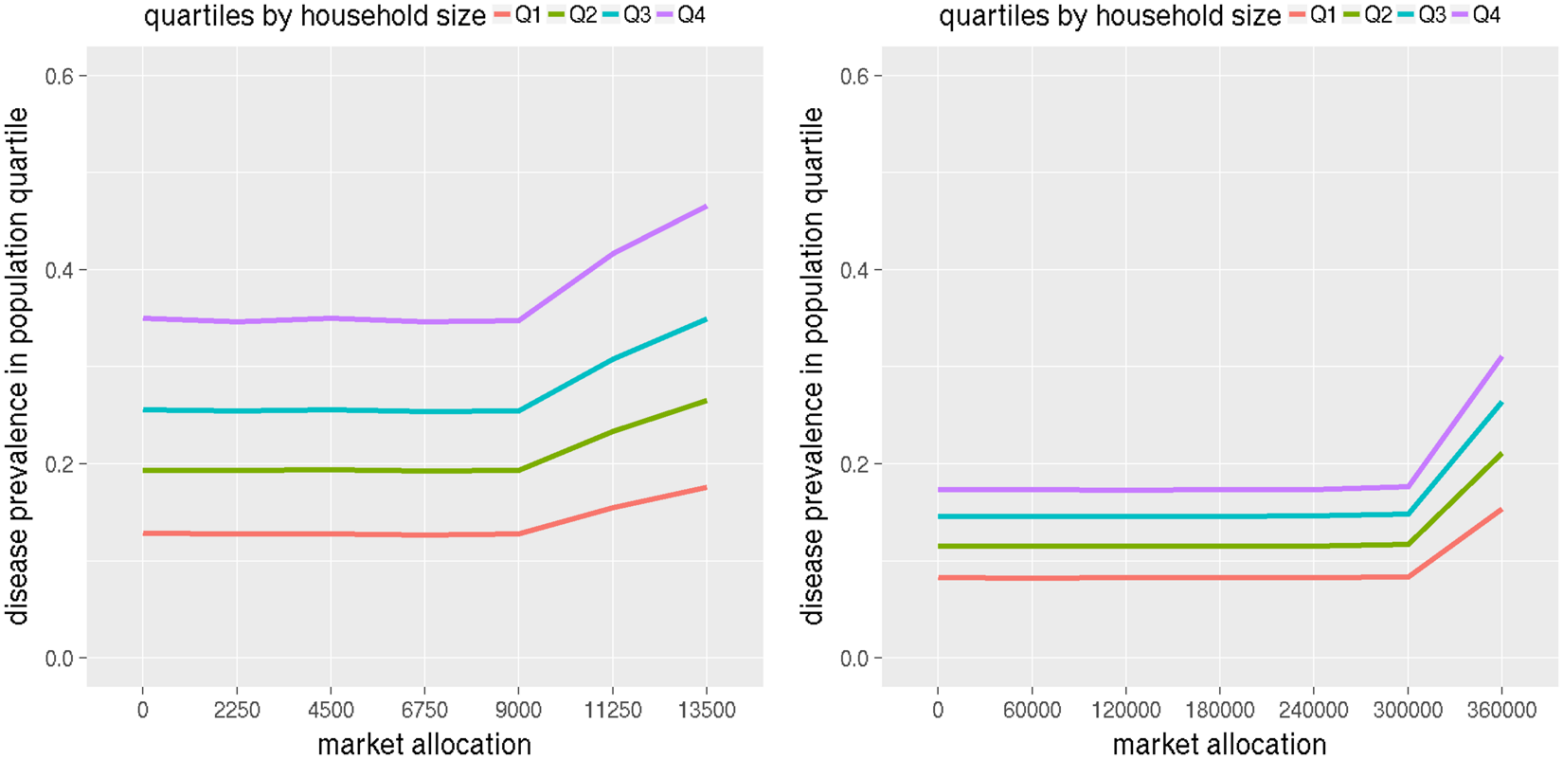
The plots show disease prevalence rates in NRV (left) and WDC (right) by household size, for different levels of antiviral allocation to the market. Note that this plot is only for the allocations made to the market (the remaining stockpile is given to the hospitals).

In this section we analyze the burden of disease by demographics and by network properties of individuals. For demographics, each region’s population is divided into quartiles by household income and by family size. Figure 7 shows infection attack rates in family size quartiles for NRV and WDC at different levels of market allocation. In both regions, bigger family size is associated with a higher level of disease prevalence at all allocation levels. This is consistent with the fact that larger families tend to have a higher number of contacts and hence encounter more pathways to transmission and a bigger burden of the disease.

The burden of disease split by income quartiles is more surprising. The findings show that infection attack rates under the optimal split of (9k, 6k) in NRV is 20%, 21.7%, 20.4%, and 16.9% for the four income quartiles respectively but the usage of market antiviral stockpile is 0, 0, 0, and 23.4% respectively. This means that although the burden of disease on the richest quartile is the least i.e. 16.9%, it is the only quartile that is able to afford antivirals from the market. 23.4% of the richest quartile are able to buy the market stockpile; the other three income quartiles get none from the market.

For WDC, similar results hold true. The infection attack rates by income quartiles in WDC for the optimal split (300k, 100k) are 14.5%, 13.3%, 12%, and 10.3% but the usage of market antiviral stockpile is 0, 0, 0, and 29.1% respectively. In WDC the burden of disease goes down by income quartile but access to the market stockpile is only restricted to the richest quartile where 29.1% of people are able to purchase it.

**Figure 8:**
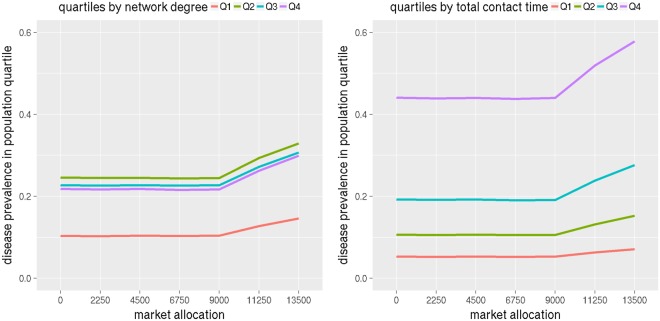
The plots show disease prevalence rates in NRV for different levels of antiviral allocations to the market (the remaining stockpile is given to the hospitals) by population quartiles. The left subfigure shows population quartiles by degree and the right one by total contact time.

Next we consider the burden of disease by subgroups based on their network properties. We calculate degree and total contact time (weighted degree) of each person in the social network and use it to divide them into four quartiles. Figure 8 shows infection attack rates in each quartile by degree and contact time in left and right subfigures respectively for NRV. The infection attack rate increases with market allocation in both plots. This is consistent with the results shown in Figs 4 and 5, where infection attack rates increased with more allocation of antivirals to markets.

However the left subfigure which splits individuals by degree, shows the second quartile has higher infection attack rates compared to the third and fourth quartiles. This implies that degree is not the most important feature in influencing infection attack rates. A person can have a high degree but the time spent with each contact may be fairly small. So we consider weighted degree or the total contact time in the right subfigure. This shows the infection attack rate consistently increases with contact time.

**Figure 9:**
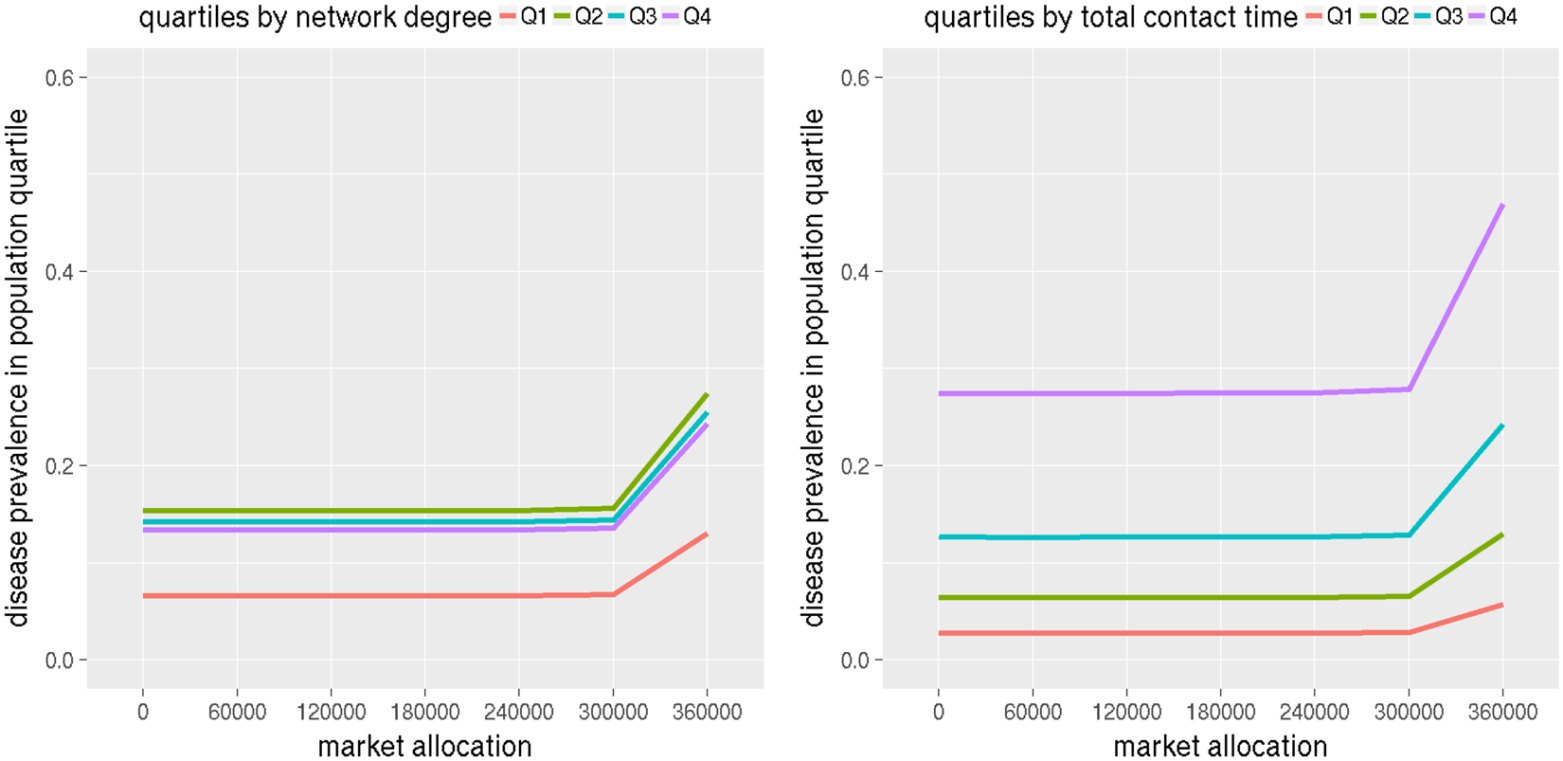
The plots show disease prevalence rates in WDC for different levels of antiviral allocations to the market (the remaining stockpile is given to the hospitals) versus population quartiles. The left subfigure shows population quartiles by degree and the right one by total contact time.

Similar results hold for WDC. Plots in Fig. 9 show infection attack rates for population quartiles split by degree and contact time. As market allocation increases and as contact time increases, infection attack rates increase.

### Wastage From Market Stockpile

**Figure 10:**
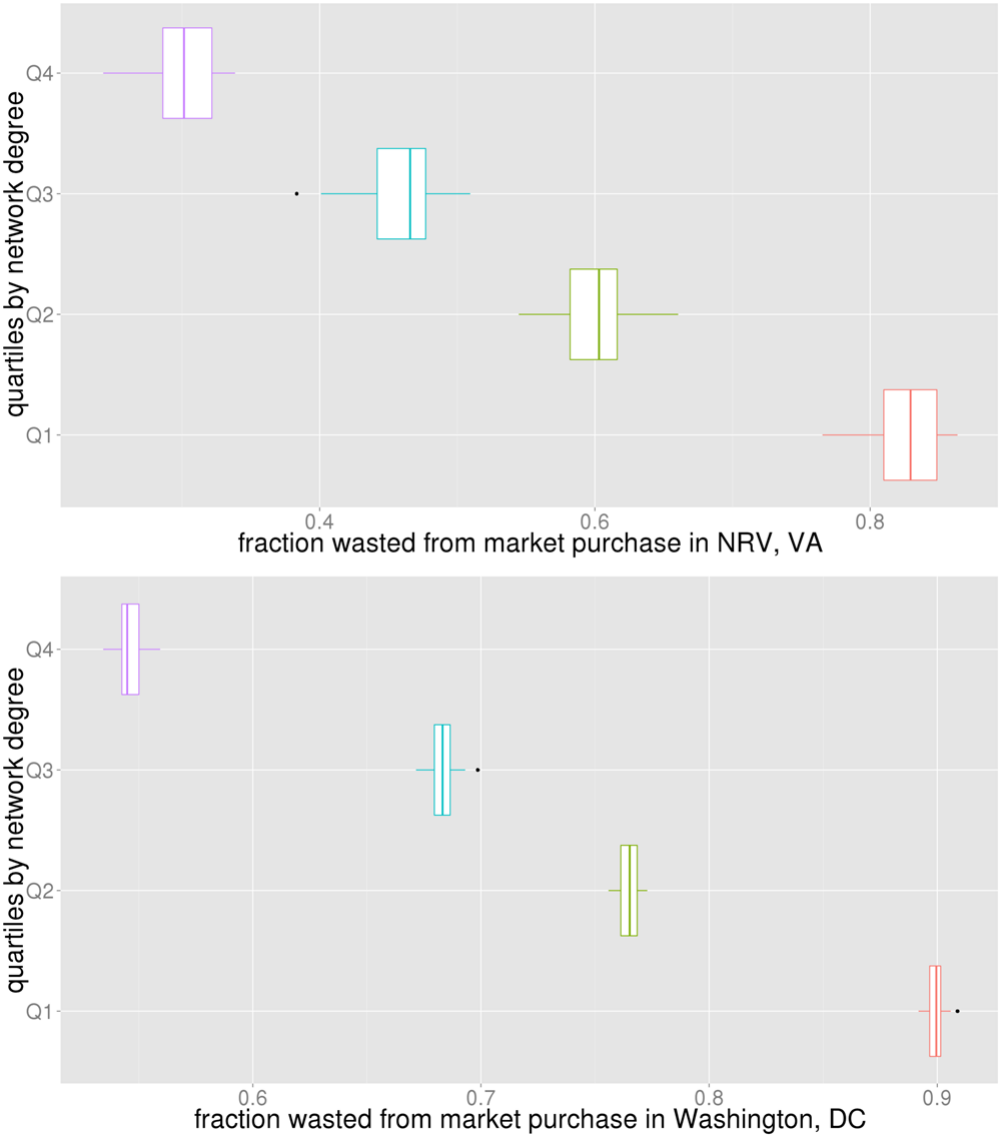
The horizontal axis shows the fraction of the antivirals that were wasted from what was bought in the market. The vertical axis shows subpopulations split into four quartiles by degree.

Next we study if the antivirals purchased from the market were effectively utilized or wasted. Box plots in Fig. 10 show the fraction of antivirals that was wasted from what was bought in the market in NRV and WDC. The vertical axis shows subpopulations split into four quartiles by degree. The higher the degree, the lower the wastage in both regions.

**Figure 11:**
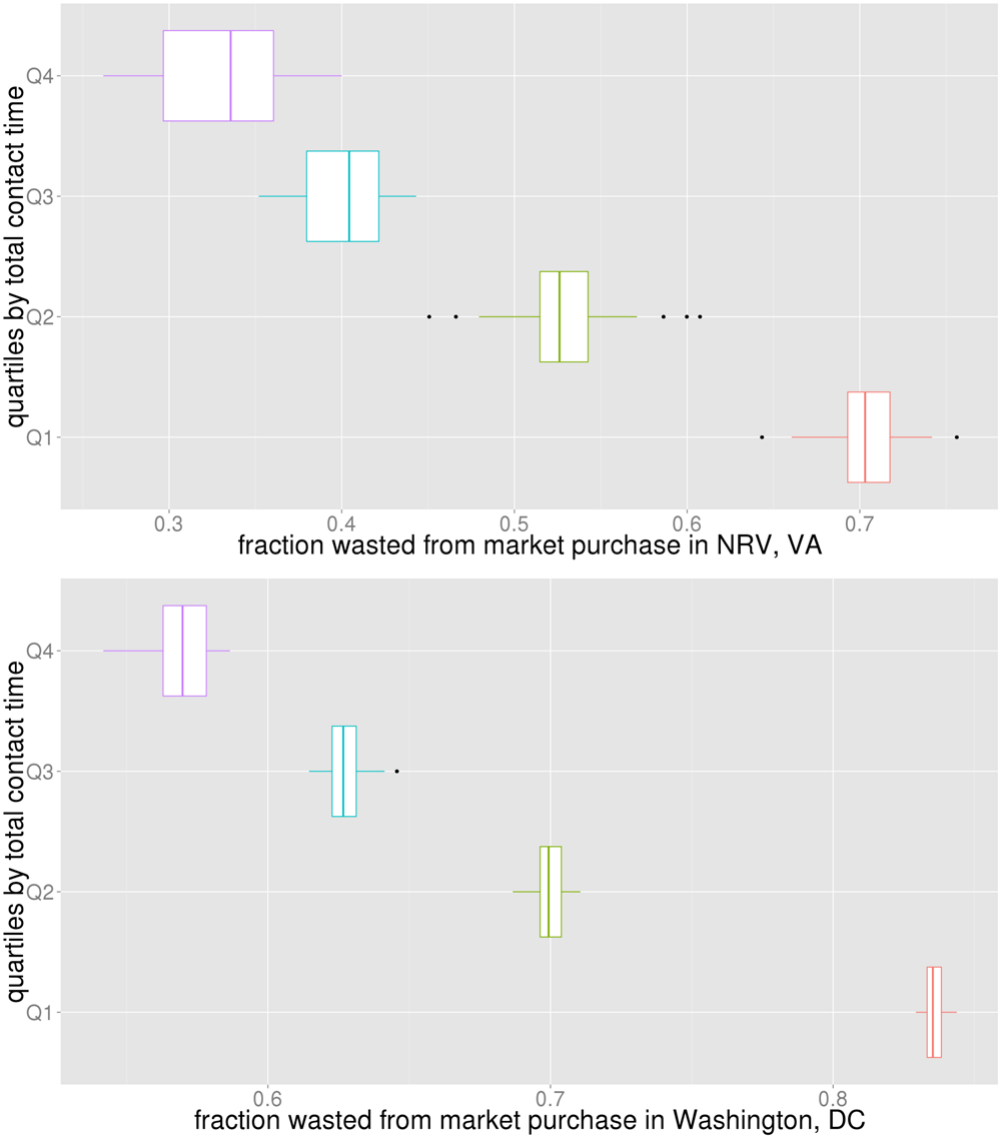
The horizontal axis shows the fraction of the antivirals that were wasted from what was bought in the market. The vertical axis shows subpopulations split into four quartiles by total contact time (weighted degree).

Box plots in Fig. 11 show this fraction for NRV and WDC by contact time quartiles. The vertical axis shows subpopulations split into four quartiles by contact time. The higher the overall contact time, the lower the wastage in both regions. If antivirals could be given to individuals who have high contact time, it will result in the least number of infections and the least wastage. If the distribution is made through the market, the price of antivirals will determine who can get it. But if antivirals could be distributed as prophylaxis by the public sector, giving it to individuals with high contact time would be more beneficial.

In the real world it is not easy to measure either individuals’ degree or their contact times. For a policy maker to use these results to make an implementable policy, it is important to tie the network properties to the demographics of the individuals. To achieve this, we consider a variety of demographics such as age, gender, income, family size etc. to determine the most correlated variable with total contact time. The results find that members of large households and school-aged children between 5 to 18 years old tend to have high contact times.

**Figure 12:**
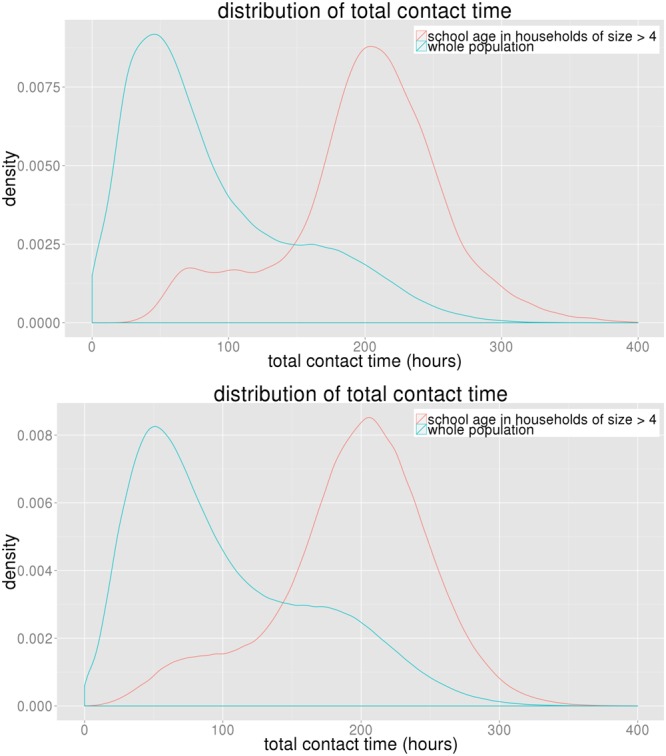
The plots show that school-aged (5 to 18 years old) children of large households have significantly higher contact time than an average person in both NRV and WDC, as shown in the upper and lower plots respectively.

Figure 12 compares the distribution of individual’s total contact time in the population with that of school-aged children from large households. We find that in both regions, school-aged children of families of size larger than 4 have much higher mean contact time than an average person in the population. This result helps connect network features with demographics so the network-based results can be implemented by public health officials.

## Sensitivity Analysis

We perform a detailed sensitivity analysis of this study. Many parameters and configurations are altered to study the robustness of the results. To address the stochasticity in epidemic dynamics, we create 30 replicates of each configuration and report the mean values. The plots show the error bars whenever possible. The box plots show the median, first and third quartile (“hinges”); the upper (lower) whisker extends to the highest (lowest) value that is within 1.5 * IQR (inter-quartile range).

Table 3 shows a detailed design of specific parameters and their values used in measuring the sensitivity. Across all these configurations, we find that epidemic size monotonically increases when greater fractions of the antiviral stockpile are given to the market. The optimal split point varies between 8,250 to 9,750 in NRV (as opposed to 9k) and between 280,000 to 320,000 in WDC (as opposed to 300k). Under no configuration, the optimal allocation results in assigning the entire stockpile to market or to hospitals.

**Table 3:** Range of parameters considered to study sensitivity of results.

Parameter	Change		Description
	From	To	
hospital antiviral compliance	0.5	0.75	increase probability that a diagnosed person actually takes the prescribed antivirals
*β* in demand function	40	120	increase elasticity of demand to disease prevalence
*P*_max_ ($)	100	75	lower the maximum market price of antivirals
threshold of “stay-home”	0.0005	0.0001	lower threshold for triggering “stay-home” intervention
compliance rate of “stay-home”	0.5	0.75	increase probability of diagnosed people taking “stay-home” measure
demand function	exponential	linear	change the functional form of the demand function
cumulative number of infections as a percentage of population	40%	50%	increase transmissibility to reach a higher infection attack rate

We found that the oscillatory behavior of the epidemic curve held for only some of the configurations. In NRV, none of the parameter settings considered resulted in a bimodal curve. We believe it is because the population of NRV is much smaller, so once the epidemic starts to die out, it does not easily get revived.

Disease prevalence always increased consistently with household size and total contact time in both regions and under all configurations. The correlation of disease prevalence with degree is less clear as shown in the experiments reported above. In all cases, the richest income quartile had the lowest infections and yet was the only quartile that was able to purchase antivirals from the market. The lower income quartiles did not want it first when the prevalence was low and the price was low. When prevalence increased, the demand increased which raised the price to the extent that lower income people could not afford to buy it. Wastage consistently decreased with degree and total contact time in all configurations. This result was robust for all parameter settings.

### Limitations

There are several limitations of this study: (*i*) it does not take into account the immunity levels of individuals; (*ii*) repeated use of prophylactic antivirals could build resistance to the drug and become ineffective; and (*iii*) several assumptions have been made regarding the range of parameters which may not hold true during an actual pandemic.

## Conclusions

This research uses a detailed individual-based, network model to study feedback between disease dynamics, interventions, individual behavior, and social contact network when a limited supply of medical resources is available and interventions are modeled as a function of disease prevalence. Results show that an optimal split of antiviral stockpile between the private and public sectors can be reached, which will contain the spread of the disease and recover the cost of antivirals entirely from the private sector.

A detailed analysis of the burden of disease, accessibility of antivirals, and the level of wastage of market stockpile by demographics and network properties has been performed. We find infections increase by household size and contact time but not necessarily by degree. Accessibility to market stockpile of antivirals is limited to individuals in the richest income quartile; and among those who purchase antivirals from the market, wastage of antivirals decreased by degree and contact time. Additionally school-aged children of large families are found to have the highest contact time compared to any other demographic, which makes them vulnerable to catching infections, as well as act as vectors of transmission, so priority should be given to them when distributing limited medical resources.

## Data Availability

The data generated and/or analyzed in the current study are available from the corresponding author upon request.
